# P038 Sequential bilateral central retinal artery occlusion despite high-dose glucocorticoid therapy in biopsy-proven giant cell arteritis

**DOI:** 10.1093/rap/rkag113.068

**Published:** 2026-11-03

**Authors:** Hiba Salih, Anupama Nandagudi

**Affiliations:** Rheumatology, Basildon University Hospital, Basildon, United Kingdom; Rheumatology, Basildon University Hospital, Basildon, United Kingdom

## Abstract

**Introduction:**

Giant cell arteritis (GCA) is the most common systemic vasculitis affecting older adults and represents a medical emergency because of the risk of irreversible visual loss. High-dose glucocorticoids usually prevent further ischaemic complications, yet visual decline may still occur despite prompt treatment. Understanding the mechanisms of treatment-refractory visual loss remains challenging, particularly when conventional markers of disease activity are limited.

We present a biopsy- and ultrasound-confirmed case of GCA in an elderly gentleman who developed sequential bilateral central retinal artery occlusion despite high-dose glucocorticoid therapy, highlighting important considerations in monitoring, imaging, and assessing disease activity in GCA.

**Case description:**

An 89-year-old man presented with acute painless visual loss in the right eye. MRI, MRA and CT brain showed no acute cerebrovascular pathology. Carotid Doppler demonstrated 50% carotid artery stenosis, and antiplatelet therapy was switched from aspirin to clopidogrel. Ophthalmological assessment confirmed right central retinal artery occlusion.

Two weeks later, he developed a pressure-like headache and two transient episodes of visual loss affecting the left eye. Temporal artery ultrasound demonstrated bilateral halo signs, and temporal artery biopsy confirmed GCA, showing chronic inflammatory cell infiltration with fragmentation of the internal and external elastic laminae. Prednisolone 60 mg daily was commenced. Inflammatory markers were only mildly elevated (CRP 10 mg/L) and normalised with treatment.

Four weeks later, despite ongoing high-dose glucocorticoid therapy, he experienced a further 30-minute episode of visual loss in the left eye. He received three doses of intravenous methylprednisolone 500 mg followed by prednisolone 60 mg daily, with minimal improvement. Ophthalmological reassessment confirmed bilateral central retinal artery occlusion. Notably, visual deterioration occurred despite treatment and without a rise in inflammatory markers.

Following multidisciplinary discussion involving rheumatology, ophthalmology, stroke physicians and tertiary vasculitis specialists, extensive investigations were undertaken to exclude alternative causes. Echocardiography and 24-hour tape revealed no cardiac source of embolism. Autoimmune screening, antiphospholipid antibody testing and thrombophilia screening were negative. Histopathological review demonstrated no evidence of cholesterol embolisation. Methotrexate was introduced as a steroid-sparing agent.

When prednisolone had been tapered to 10 mg daily, repeat temporal artery ultrasound demonstrated persistent bilateral vessel wall inflammation with residual halo signs. Methotrexate was discontinued, azathioprine initiated, and prednisolone increased to 40 mg daily. Repeat ultrasound six weeks later demonstrated complete resolution of the halo sign. He remained in clinical remission with normal inflammatory markers and no further visual deterioration but was left with permanent visual impairment and loss of independence.

**Discussion:**

Visual loss remains one of the most feared complications of GCA and is associated with significant morbidity. Permanent visual impairment occurs in up to 20% of patients and may result from anterior ischaemic optic neuropathy, central retinal artery occlusion (CRAO), cilioretinal artery occlusion, ocular ischaemic syndrome or, less commonly, posterior ischaemic optic neuropathy. Prompt initiation of high-dose glucocorticoids reduces the risk of further ischaemic events. However, this case illustrates that visual deterioration may still progress despite appropriate therapy.

Our patient developed sequential bilateral CRAO despite treatment with prednisolone 60 mg daily and subsequent intravenous methylprednisolone. His initial presentation with isolated visual symptoms and few systemic features highlights the diagnostic challenges of atypical GCA. Several mechanisms were considered. First, irreversible vascular injury may have occurred before treatment initiation, leading to progression despite adequate immunosuppression. Second, an alternative vascular process, such as embolic disease, was explored. However, extensive investigations—including vascular imaging, cardiac assessment, thrombophilia screening and antiphospholipid antibody testing—did not identify another cause. Third, the case raises the possibility that vascular inflammation may persist despite normal inflammatory markers, underscoring the limitations of relying solely on symptoms, CRP and ESR to assess disease activity.

A further challenge was the persistence of bilateral halo signs on temporal artery ultrasound despite prolonged glucocorticoid therapy and methotrexate. Ongoing imaging abnormalities, combined with clinical uncertainty, informed the decision to switch to azathioprine. Subsequent resolution of the halo sign suggests that vascular ultrasound may provide information about persistent disease activity and guide therapeutic decisions when clinical assessment and inflammatory markers are incongruent.

This case highlights the complexity of managing refractory or atypical GCA and emphasises the need for evaluation of patients who continue to develop ischaemic complications despite standard treatment. It also supports an emerging role for vascular ultrasound in monitoring disease activity beyond conventional biomarkers.

**Key learning points:**

• Visual loss may be the presenting feature of giant cell arteritis and can occur in the absence of significant systemic symptoms or markedly elevated inflammatory markers.

• Prompt initiation of high-dose glucocorticoids remains essential in suspected GCA; however, ischaemic complications may still occur despite appropriate treatment.

• Sequential visual loss despite treatment should prompt reconsideration of the diagnosis, investigation for alternative vascular mechanisms and multidisciplinary review.

• Normal or near-normal CRP and ESR do not necessarily exclude ongoing vascular inflammation, particularly in patients with persistent symptoms or concerning imaging findings.

• Temporal artery ultrasound may provide additional information when clinical assessment and inflammatory markers are inconclusive and may have a role in monitoring disease activity.

• Persistent bilateral halo signs despite glucocorticoid therapy and methotrexate raised uncertainty regarding ongoing disease activity and influenced treatment adjustment.

